# Supplementary material for: Increasing the willingness to participate in organ donation through humorous health communication: (Quasi-) experimental evidence
Source: PLoS One. 2020 Nov 20;15(11):e0241208. doi: 10.1371/journal.pone.0241208 (PMC7678957; doi:10.1371/journal.pone.0241208)
Supplement: S1 Table — n = 3,510. Treatment: 0 = control group without topic of organ donation, 1 = intervention group with organ donation stand-up. Attitude: mean across seven items, ranging from 1 to 7. Perceived funniness: 1 = not humorous to 10 = humorous. 95% BC CI: corrected 95% confidence interval with lower and upper border, based on 5,000 bootstrap resamples, CIs that do not contain zero indicate a significant indirect effect with p < .05. (DOCX) [file pone.0241208.s002.docx]

S1 Table (corresponding to Figure 2A, Study 1)

*Mediation analysis: Effect of treatment (X) on attitude T2 (Y) via perceived funniness (M), model 4 (Hayes, 2013).*

|  | Mediator variable model (outcome: perceived funniness) | | |  |
| --- | --- | --- | --- | --- |
| Predictor | *B* | SE | 95% CI | *p* |
| Constant | 7.3234 | 0.0514 | (8.2226, 8.4243) | <.001 |
| Treatment | -0.5630 | 0.0597 | (-0.6801, -0.4458) | <.001 |
|  | Dependent variable model (outcome: attitude T2) | | | |
|  | Model summary: R^2^ = 0.0546 | | |  |
| Predictor | *B* | SE | 95% CI | *p* |
| Constant | 5.0581 | 0.0878 | (4.8860, 5.2302) | <.001 |
| Treatment | 0.3645 | 0.0355 | (0.2949, 0.4340) | <.001 |
| Perceived funniness | 0.1123 | 0.0099 | (0.0928, 0.1317) | <.001 |
|  | Indirect effect of X on Y via perceived funniness | | |  |
| Mediator | *B* | SE | 95% BC CI |  |
| Perceived funniness | -0.0632 | 0.0087 | (-0.0810, -0.0471) |  |

*n* = 3,510

Treatment: 0 = control group without topic of organ donation, 1 = intervention group with organ donation stand-up. Attitude: mean across seven items, ranging from 1 to 7. Perceived funniness: 1 = not humorous to 10 = humorous. 95% BC CI: corrected 95% confidence interval with lower and upper border, based on 5,000 bootstrap resamples, CIs that do not contain zero indicate a significant indirect effect with *p* < .05.
