# Supplementary material for: Increasing the willingness to participate in organ donation through humorous health communication: (Quasi-) experimental evidence
Source: PLoS One. 2020 Nov 20;15(11):e0241208. doi: 10.1371/journal.pone.0241208 (PMC7678957; doi:10.1371/journal.pone.0241208)
Supplement: S2 Table — n = 2,444. Treatment: 0 = control group without topic of organ donation, 1 = intervention group with organ donation stand-up. Intention: mean across three items, ranging from 1 to 7. Perceived funniness: 1 = not humorous to 10 = humorous. 95% BC CI: corrected 95% confidence interval with lower and upper border, based on 5,000 bootstrap resamples, CIs that do not contain zero indicate a significant indirect effect with p < .05. (DOCX) [file pone.0241208.s003.docx]

S2 Table (corresponding to Figure 2A, Study 1)

*Mediation analysis: Effect of treatment (X) on intention T2 (Y) via perceived funniness (M), model 4 (Hayes, 2013).*

|  | Mediator variable model (outcome: perceived funniness) | | |  |
| --- | --- | --- | --- | --- |
| Predictor | *B* | SE | 95% CI | *p* |
| Constant | 8.2896 | 0.0653 | (8.1616, 8.4175) | <.001 |
| Treatment | -0.6042 | 0.0750 | (-0.7513, -0.4571) | <.001 |
|  | Dependent variable model (outcome: intention T2) | | | |
|  | Model summary: R^2^ = 0.0591 | | |  |
| Predictor | *B* | SE | 95% CI | *p* |
| Constant | 2.9661 | 0.1810 | (2.6111, 3.3210) | <.001 |
| Treatment | 0.7283 | 0.0764 | (0.5785, 0.8782) | <.001 |
| Perceived funniness | 0.1900 | 0.0203 | (0.1501, 0.2299) | <.001 |
|  | Indirect effect of X on Y via perceived funniness | | |  |
| Mediator | *B* | SE | 95% BC CI |  |
| Perceived funniness | -0.1148 | 0.0197 | (-0.1559, -0.0787) |  |

*n* = 2,444

Treatment: 0 = control group without topic of organ donation, 1 = intervention group with organ donation stand-up. Intention: mean across three items, ranging from 1 to 7. Perceived funniness: 1 = not humorous to 10 = humorous. 95% BC CI: corrected 95% confidence interval with lower and upper border, based on 5,000 bootstrap resamples, CIs that do not contain zero indicate a significant indirect effect with *p* < .05.
