# Supplementary material for: Increasing the willingness to participate in organ donation through humorous health communication: (Quasi-) experimental evidence
Source: PLoS One. 2020 Nov 20;15(11):e0241208. doi: 10.1371/journal.pone.0241208 (PMC7678957; doi:10.1371/journal.pone.0241208)
Supplement: S3 Table — n = 3,504. Treatment: 0 = control group without topic of organ donation, 1 = intervention group with organ donation stand-up. Attitude: mean across seven items, ranging from 1 to 7. Perceived funniness: 1 = not humorous to 10 = humorous. 95% BC CI: corrected 95% confidence interval with lower and upper border, based on 5,000 bootstrap resamples, CIs that do not contain zero indicate a significant indirect effect with p < .05. (DOCX) [file pone.0241208.s004.docx]

S3 Table (corresponding to Figure 2A, Study 1)

*Mediation analysis: Effect of treatment (X) on attitude T2 (Y) via perceived funniness (M), controlled for the attitude T1 (covariate), model 4 (Hayes, 2013).*

|  | Mediator variable model (outcome: perceived funniness) | | |  |
| --- | --- | --- | --- | --- |
| Predictor | *B* | SE | 95% CI | *p* |
| Constant | 7.0339 | 0.1678 | (6.7050, 7.3629) | <.001 |
| Treatment | -0.5696 | 0.0592 | (-0.6857, -0.4536) | <.001 |
| Attitude T1 | 0.2168 | 0.0268 | (0.1643, 0.2694) | <.001 |
|  | Dependent variable model (outcome: attitude T2) | | | |
|  | Model summary: R^2^ = 0.6088 | | |  |
| Predictor | *B* | SE | 95% CI | *p* |
| Constant | 1.2455 | 0.0783 | (1.0920, 1.3990) | <.001 |
| Treatment | 0.3246 | 0.0228 | (0.2798, 0.3693) | <.001 |
| Perceived funniness | 0.0506 | 0.0064 | (0.0380, 0.0632) | <.001 |
| Attitude T1 | 0.7247 | 0.0103 | (0.7045, 0.7448) | <.001 |
|  | Indirect effect of X on Y via perceived funniness | | |  |
| Mediator | *B* | SE | 95% BC CI |  |
| Perceived funniness | -0.0288 | 0.0049 | (-0.0392, -0.0195) |  |

*n* = 3,504

Treatment: 0 = control group without topic of organ donation, 1 = intervention group with organ donation stand-up. Attitude: mean across seven items, ranging from 1 to 7. Perceived funniness: 1 = not humorous to 10 = humorous. 95% BC CI: corrected 95% confidence interval with lower and upper border, based on 5,000 bootstrap resamples, CIs that do not contain zero indicate a significant indirect effect with *p* < .05.
