# Supplementary material for: Increasing the willingness to participate in organ donation through humorous health communication: (Quasi-) experimental evidence
Source: PLoS One. 2020 Nov 20;15(11):e0241208. doi: 10.1371/journal.pone.0241208 (PMC7678957; doi:10.1371/journal.pone.0241208)
Supplement: S4 Table — n = 2,379. Treatment: 0 = control group without topic of organ donation, 1 = intervention group with organ donation stand-up. Intention: mean across three items, ranging from 1 to 7. Perceived funniness: 1 = not humorous to 10 = humorous. 95% BC CI: corrected 95% confidence interval with lower and upper border, based on 5,000 bootstrap resamples, CIs that do not contain zero indicate a significant indirect effect with p < .05. (DOCX) [file pone.0241208.s005.docx]

S4 Table (corresponding to Figure 2A, Study 1)

*Mediation analysis: Effect of treatment (X) on intention T2 (Y) via perceived funniness (M), controlled for the intention T1 (covariate), model 4 (Hayes, 2013).*

|  | Mediator variable model (outcome: perceived funniness) | | |  |
| --- | --- | --- | --- | --- |
| Predictor | *B* | SE | 95% CI | *p* |
| Constant | 7.7178 | 0.1067 | (7.5086, 7.9269) | <.001 |
| Treatment | -0.6341 | 0.0746 | (-0.7805, -0.4878) | <.001 |
| Intention T1 | 0.1374 | 0.0196 | (0.0989, 0.1759) | <.001 |
|  | Dependent variable model (outcome: intention T2) | | | |
|  | Model summary: R^2^ = 0.5970 | | |  |
| Predictor | *B* | SE | 95% CI | *p* |
| Constant | 0.6906 | 0.1261 | (0.4433, 0.9379) | <.001 |
| Treatment | 0.5676 | 0.0500 | (0.4695, 0.6657) | <.001 |
| Perceived funniness | 0.0816 | 0.0136 | (0.0551, 0.1082) | <.001 |
| Intention T1 | 0.7382 | 0.0131 | (0.7125, 0.7639) | <.001 |
|  | Indirect effect of X on Y via perceived funniness | | |  |
| Mediator | *B* | SE | 95% BC CI |  |
| Perceived funniness | -0.0518 | 0.0136 | (-0.0803, -0.0270) |  |

*n* = 2,379

Treatment: 0 = control group without topic of organ donation, 1 = intervention group with organ donation stand-up. Intention: mean across three items, ranging from 1 to 7. Perceived funniness: 1 = not humorous to 10 = humorous. 95% BC CI: corrected 95% confidence interval with lower and upper border, based on 5,000 bootstrap resamples, CIs that do not contain zero indicate a significant indirect effect with *p* < .05.
