# Supplementary material for: Increasing the willingness to participate in organ donation through humorous health communication: (Quasi-) experimental evidence
Source: PLoS One. 2020 Nov 20;15(11):e0241208. doi: 10.1371/journal.pone.0241208 (PMC7678957; doi:10.1371/journal.pone.0241208)
Supplement: S5 Table — n = 144. Treatment: 0 = neutral control treatment, 1 = humorous treatment. Attitude: mean across three items, ranging from 1 to 7. Perceived funniness: mean across four items, ranging from 1 to 7. 95% BC CI: corrected 95% confidence interval with lower and upper border, based on 5,000 bootstrap resamples, CIs that do not contain zero indicate a significant indirect effect with p < .05. (DOCX) [file pone.0241208.s006.docx]

S5 Table (corresponding to Figure 2A, Study 2)

*Mediation analysis: Effect of treatment (X) on attitude T2 (Y) via perceived funniness (M), model 4 (Hayes, 2013).*

|  | Mediator variable model (outcome: perceived funniness) | | |  |
| --- | --- | --- | --- | --- |
| Predictor | *B* | SE | 95% CI | *p* |
| Constant | 2.2917 | .1396 | (2.0158, 2.5675) | <.001 |
| Treatment | 2.9444 | .1974 | (2.5543, 3.3346) | <.001 |
|  | Dependent variable model (outcome: attitude T2) | | | |
|  | Model summary: R^2^ = 0.0284 | | |  |
| Predictor | *B* | SE | 95% CI | *p* |
| Constant | 6.0138 | 0.1544 | (5.7085, 6.3191) | <.001 |
| Treatment | -0.2167 | 0.2055 | (-0.6231, 0.1896) | .2935 |
| Perceived funniness | 0.1039 | 0.0545 | (-0.0039, 0.2118) | .0588 |
|  | Indirect effect of X on Y via perceived funniness | | |  |
| Mediator | *B* | SE | 95% BC CI |  |
| Perceived funniness | 0.3060 | 0.1780 | (-0.0634, 0.6398) |  |

*n* = 144

Treatment: 0 = neutral control treatment, 1 = humorous treatment. Attitude: mean across three items, ranging from 1 to 7. Perceived funniness: mean across four items, ranging from 1 to 7. 95% BC CI: corrected 95% confidence interval with lower and upper border, based on 5,000 bootstrap resamples, CIs that do not contain zero indicate a significant indirect effect with *p* < .05.
