# Supplementary material for: Increasing the willingness to participate in organ donation through humorous health communication: (Quasi-) experimental evidence
Source: PLoS One. 2020 Nov 20;15(11):e0241208. doi: 10.1371/journal.pone.0241208 (PMC7678957; doi:10.1371/journal.pone.0241208)
Supplement: S6 Table — n = 90. Treatment: 0 = neutral control treatment, 1 = humorous treatment. Intention: mean across three items, ranging from 1 to 7. Perceived funniness: mean across four items, ranging from 1 to 7. 95% BC CI: corrected 95% confidence interval with lower and upper border, based on 5,000 bootstrap resamples, CIs that do not contain zero indicate a significant indirect effect with p < .05. (DOCX) [file pone.0241208.s007.docx]

S6 Table (corresponding to Figure 2A, Study 2)

*Mediation analysis: Effect of treatment (X) on intention T2 (Y) via perceived funniness (M), model 4 (Hayes, 2013).*

|  | Mediator variable model (outcome: perceived funniness) | | |  |
| --- | --- | --- | --- | --- |
| Predictor | *B* | SE | 95% CI | *p* |
| Constant | 2.3023 | 0.1922 | (1.9205, 2.6842) | <.001 |
| Treatment | 2.8892 | 0.2659 | (2.3607, 3.4176) | <.001 |
|  | Dependent variable model (outcome: intention T2) | | | |
|  | Model summary: R^2^ = 0.0551 | | |  |
| Predictor | *B* | SE | 95% CI | *p* |
| Constant | 4.4231 | 0.3770 | (3.6737, 5.1724) | <.001 |
| Treatment | -0.3342 | 0.4921 | (-1.3123, 0.6440) | .4990 |
| Perceived funniness | 0.2472 | 0.1289 | (-0.0091, 0.5035) | .0585 |
|  | Indirect effect of X on Y via perceived funniness | | |  |
| Mediator | *B* | SE | 95% BC CI |  |
| Perceived funniness | 0.7142 | 0.4370 | (-0.1968, 1.5284) |  |

*n* = 90

Treatment: 0 = neutral control treatment, 1 = humorous treatment. Intention: mean across three items, ranging from 1 to 7. Perceived funniness: mean across four items, ranging from 1 to 7. 95% BC CI: corrected 95% confidence interval with lower and upper border, based on 5,000 bootstrap resamples, CIs that do not contain zero indicate a significant indirect effect with *p* < .05.
