# Supplementary material for: Increasing the willingness to participate in organ donation through humorous health communication: (Quasi-) experimental evidence
Source: PLoS One. 2020 Nov 20;15(11):e0241208. doi: 10.1371/journal.pone.0241208 (PMC7678957; doi:10.1371/journal.pone.0241208)
Supplement: S7 Table — n = 144 Treatment: 0 = neutral control treatment, 1 = humorous treatment. Attitude: mean across seven items, ranging from 1 to 7. Perceived funniness: mean across four items, ranging from 1 to 7. 95% BC CI: corrected 95% confidence interval with lower and upper border, based on 5,000 bootstrap resamples, CIs that do not contain zero indicate a significant indirect effect with p < .05. (DOCX) [file pone.0241208.s008.docx]

S7 Table (corresponding to Figure 2A, Study 2)

*Mediation analysis: Effect of treatment (X) on attitude T2 (Y) via perceived funniness (M), controlled for the attitude T1 (covariate), model 4 (Hayes, 2013).*

|  | Mediator variable model (outcome: perceived funniness) | | |  |
| --- | --- | --- | --- | --- |
| Predictor | *B* | SE | 95% CI | *p* |
| Constant | 1.4195 | 0.7082 | (0.0194, 2.8196) | .0469 |
| Treatment | 2.9261 | 0.1975 | (2.5357, 3.3165) | <.001 |
| Attitude T1 | 0.1497 | 0.1177 | (-0.0849, 0.3806) | .2112 |
|  | Dependent variable model (outcome: attitude T2) | | | |
|  | Model summary: R^2^ = 0.7046 | | |  |
| Predictor | *B* | SE | 95% CI | *p* |
| Constant | 1.6399 | 0.2589 | (1.1281, 2.1516) | <.001 |
| Treatment | -0.1432 | 0.1138 | (-0.3682, 0.0818) | .2104 |
| Perceived funniness | 0.0468 | 0.0304 | (-0.0132, 0.1068) | .1256 |
| Attitude T1 | 0.7638 | 0.0427 | (0.6794, 0.8481) | <.001 |
|  | Indirect effect of X on Y via perceived funniness | | |  |
| Mediator | *B* | SE | 95% BC CI |  |
| Perceived funniness | 0.1369 | 0.1117 | (-0.0887, 0.3450) |  |

*n* = 144

Treatment: 0 = neutral control treatment, 1 = humorous treatment. Attitude: mean across seven items, ranging from 1 to 7. Perceived funniness: mean across four items, ranging from 1 to 7. 95% BC CI: corrected 95% confidence interval with lower and upper border, based on 5,000 bootstrap resamples, CIs that do not contain zero indicate a significant indirect effect with *p* < .05.
