# Supplementary material for: Increasing the willingness to participate in organ donation through humorous health communication: (Quasi-) experimental evidence
Source: PLoS One. 2020 Nov 20;15(11):e0241208. doi: 10.1371/journal.pone.0241208 (PMC7678957; doi:10.1371/journal.pone.0241208)
Supplement: S8 Table — n = 86. Treatment: 0 = neutral control treatment, 1 = humorous treatment. Intention: mean across three items, ranging from 1 to 7. Perceived funniness: mean across four items, ranging from 1 to 7 95% BC CI: corrected 95% confidence interval with lower and upper border, based on 5,000 bootstrap resamples, CIs that do not contain zero indicate a significant indirect effect with p < .05. (DOCX) [file pone.0241208.s009.docx]

S8 Table (corresponding to Figure 2A, Study 2)

*Mediation analysis: Effect of treatment (X) on intention T2 (Y) via perceived funniness (M), controlled for the intention T1 (covariate), model 4 (Hayes, 2013).*

|  | Mediator variable model (outcome: perceived funniness) | | |  |
| --- | --- | --- | --- | --- |
| Predictor | *B* | SE | 95% CI | *p* |
| Constant | 1.7278 | 0.4449 | (0.8428, 2.6127) | .0002 |
| Treatment | 2.8110 | 0.2771 | (2.2598, 3.3623) | <.001 |
| Intention T1 | 0.1350 | 0.0897 | (-0.0434, 0.3133) | .1361 |
|  | Dependent variable model (outcome: intention) | | | |
|  | Model summary: R^2^ = 0.6661 | | |  |
| Predictor | *B* | SE | 95% CI | *p* |
| Constant | 1.1640 | 0.3460 | (0.4757, 1.8523) | .0012 |
| Treatment | -0.1265 | 0.2967 | (-0.7168, 0.4637) | .6709 |
| Perceived funniness | 0.1002 | 0.0785 | (-0.0560, 0.2564) | .2055 |
| Intention T1 | 0.7898 | 0.0650 | (0.6604, 0.9191) | <.001 |
|  | Indirect effect of X on Y via perceived funniness | | |  |
| Mediator | *B* | SE | 95% BC CI |  |
| Perceived funniness | 0.2817 | 0.2171 | (-0.0974, 0.7827) |  |

*n* = 86

Treatment: 0 = neutral control treatment, 1 = humorous treatment. Intention: mean across three items, ranging from 1 to 7. Perceived funniness: mean across four items, ranging from 1 to 7 95% BC CI: corrected 95% confidence interval with lower and upper border, based on 5,000 bootstrap resamples, CIs that do not contain zero indicate a significant indirect effect with *p* < .05.
