# Supplementary material for: Increasing the willingness to participate in organ donation through humorous health communication: (Quasi-) experimental evidence
Source: PLoS One. 2020 Nov 20;15(11):e0241208. doi: 10.1371/journal.pone.0241208 (PMC7678957; doi:10.1371/journal.pone.0241208)
Supplement: S9 Table — n = 144. Attitude: mean across seven items, ranging from 1 to 7. Perceived funniness: mean across four items, ranging from 1 to 7. Reactance: mean across three items, ranging from 1 to 7. 95% BC CI: corrected 95% confidence interval with lower and upper border, based on 5,000 bootstrap resamples, CIs that do not contain zero indicate a significant indirect effect with p < .05. (DOCX) [file pone.0241208.s010.docx]

S9 Table (corresponding to Figure 2B, Study 2)

*Mediation analysis: Effect of treatment (X) on attitude T2 (Y) via perceived funniness (M1) and reactance (M2), model 6 (Hayes, 2013).*

|  | Mediator variable model (outcome: perceived funniness) | | |  |
| --- | --- | --- | --- | --- |
| Predictor | *B* | SE | 95% CI | *p* |
| Constant | 2.2917 | 0.1396 | (2.0158, 2.5675) | <.001 |
| Treatment | 2.9444 | 0.1974 | (2.5543, 3.3346) | <.001 |
|  | Mediator variable model (outcome: reactance) | | |  |
| Predictor | *B* | SE | 95% CI | *p* |
| Constant | 2.2445 | 0.2340 | (1.7819, 2.7072) | <.001 |
| Treatment | 0.5747 | 0.3115 | (-0.0410, 1.1904) | .0671 |
| Perceived funniness | -0.1229 | 0.0826 | (-0.2863, 0.0405) | .1394 |
|  | Dependent variable model (outcome: attitude T2) | | | |
|  | Model summary: R^2^ = 0.2788 | | |  |
| Predictor | *B* | SE | 95% CI | *p* |
| Constant | 6.7658 | 0.1717 | (6.4264, 7.1052) | <.001 |
| Treatment | -0.0242 | 0.1799 | (-0.3797, 0.03314) | .8933 |
| Perceived funniness | 0.0628 | 0.0475 | (-0.0312, 0.1567) | .1888 |
| Reactance | -0.3350 | 0.0481 | (-0.4300, -0.2400) | <.001 |
|  | Indirect effect of X on Y via perceived funniness | | |  |
| Mediator | *B* | SE | 95% BC CI |  |
| Perceived funniness | 0.1848 | 0.1806 | (-0.1771, 0.5263) |  |
|  | Indirect effect of X on Y via reactance | | |  |
| Mediator | *B* | SE | 95% BC CI |  |
| Reactance | -0.1925 | 0.1048 | (-0.4122, -0.0034) |  |
|  | Indirect effect of X on Y via perceived funniness and reactance | | |  |
| Mediator | *B* | SE | 95% BC CI |  |
| Perceived funniness and reactance | 0.1212 | 0.0805 | (-0.0271, 0.2915) |  |

*n* = 144

Attitude: mean across seven items, ranging from 1 to 7. Perceived funniness: mean across four items, ranging from 1 to 7. Reactance: mean across three items, ranging from 1 to 7. 95% BC CI: corrected 95% confidence interval with lower and upper border, based on 5,000 bootstrap resamples, CIs that do not contain zero indicate a significant indirect effect with *p* < .05.
