# Supplementary material for: Increasing the willingness to participate in organ donation through humorous health communication: (Quasi-) experimental evidence
Source: PLoS One. 2020 Nov 20;15(11):e0241208. doi: 10.1371/journal.pone.0241208 (PMC7678957; doi:10.1371/journal.pone.0241208)
Supplement: S10 Table — n = 144. Attitude: mean across seven items, ranging from 1 to 7. Perceived funniness: mean across four items, ranging from 1 to 7. Counter-arguing: single item, ranging from 1 to 7. 95% BC CI: corrected 95% confidence interval with lower and upper border, based on 5,000 bootstrap resamples, CIs that do not contain zero indicate a significant indirect effect with p < .05. (DOCX) [file pone.0241208.s011.docx]

S10 Table (corresponding to Figure 2B, Study 2)

*Mediation analysis: Effect of treatment (X) on attitude T2 (Y) via perceived funniness (M1) and counter-arguing (M2), model 6 (Hayes, 2013).*

|  | Mediator variable model (outcome: perceived funniness) | | |  |
| --- | --- | --- | --- | --- |
| Predictor | *B* | SE | 95% CI | *p* |
| Constant | 2.2917 | 0.1396 | (2.0158, 2.5675) | <.001 |
| Treatment | 2.9444 | 0.1974 | (2.5543, 3.3346) | <.001 |
|  | Mediator variable model (outcome: counter-arguing) | | |  |
| Predictor | *B* | SE | 95% CI | *p* |
| Constant | 3.8396 | 0.3235 | (3.2001, 4.4792) | <.001 |
| Treatment | 1.1763 | 0.4305 | (0.3251, 2.0275) | .0071 |
| Perceived funniness | -0.2391 | 0.1143 | (-0.4650, -0.0132) | .0382 |
|  | Dependent variable model (outcome: attitude T2) | | | |
|  | Model summary: R^2^ = 0.1223 | | |  |
| Predictor | *B* | SE | 95% CI | *p* |
| Constant | 6.5838 | 0.2083 | (6.1720, 6.9956) | <.001 |
| Treatment | -0.0421 | 0.2012 | (-0.4398, 0.3556) | .8345 |
| Perceived funniness | 0.0684 | 0.0528 | (-0.0360, 0.1726) | .1973 |
| Counter-arguing | -0.1484 | 0.0383 | (-0.2243, -0.0726) | .0002 |
|  | Indirect effect of X on Y via perceived funniness | | |  |
| Mediator | *B* | SE | 95% BC CI |  |
| Perceived funniness | 0.2015 | 0.1832 | (-0.1615, 0.5483) |  |
|  | Indirect effect of X on Y via counter-arguing | | |  |
| Mediator | *B* | SE | 95% BC CI |  |
| Counter-arguing | -0.1746 | 0.0778 | (-0.3346, -0.0296) |  |
|  | Indirect effect of X on Y via perceived funniness and counter-arguing | | |  |
| Mediator | *B* | SE | 95% BC CI | *B* |
| Perceived funniness and counter-arguing | 0.1045 | 0.0590 | (-0.0022, 0.2299) |  |

*n* = 144

Attitude: mean across seven items, ranging from 1 to 7. Perceived funniness: mean across four items, ranging from 1 to 7. Counter-arguing: single item, ranging from 1 to 7. 95% BC CI: corrected 95% confidence interval with lower and upper border, based on 5,000 bootstrap resamples, CIs that do not contain zero indicate a significant indirect effect with *p* < .05.
