# Supplementary material for: Increasing the willingness to participate in organ donation through humorous health communication: (Quasi-) experimental evidence
Source: PLoS One. 2020 Nov 20;15(11):e0241208. doi: 10.1371/journal.pone.0241208 (PMC7678957; doi:10.1371/journal.pone.0241208)
Supplement: S11 Table — n = 90. Intention: mean across three items, ranging from 1 to 7. Perceived funniness: mean across four items, ranging from 1 to 7. Reactance: mean across three items, ranging from 1 to 7. 95% BC CI: corrected 95% confidence interval with lower and upper border, based on 5,000 bootstrap resamples, CIs that do not contain zero indicate a significant indirect effect with p < .05. (DOCX) [file pone.0241208.s012.docx]

S11 Table (corresponding to Figure 2B, Study 2)

*Mediation analysis: Effect of treatment (X) on intention T2 (Y) via perceived funniness (M1) and reactance (M2), model 6 (Hayes, 2013).*

|  | Mediator variable model (outcome: perceived funniness) | | |  |
| --- | --- | --- | --- | --- |
| Predictor | *B* | SE | 95% CI | *p* |
| Constant | 2.3023 | 0.1922 | (1.9205, 2.6842) | <.001 |
| Treatment | 2.8892 | 0.2659 | (2.3607, 3.4176) | <.001 |
|  | Mediator variable model (outcome: reactance) | | |  |
| Predictor | *B* | SE | 95% CI | *p* |
| Constant | 2.3982 | 0.2972 | (1.8076, 2.9888) | <.001 |
| Treatment | 0.5609 | 0.3879 | (-02101, 1.3319) | .1517 |
| Perceived funniness | -0.1123 | 0.1016 | (-0.3143, 0.0896) | .2720 |
|  | Dependent variable model (outcome: intention T2) | | | |
|  | Model summary: R^2^ = 0.1787 | | |  |
| Predictor | *B* | SE | 95% CI | *p* |
| Constant | 5.5234 | 0.4675 | (4.5940, 6.4527) | <.001 |
| Treatment | -0.0768 | 0.4679 | (-1.0052, 0.8516) | .8696 |
| Perceived funniness | 0.1957 | 0.1218 | (-0.0464, 0.4377) | .1117 |
| Reactance | -0.4588 | 0.1276 | (-0.7124, -0.2052) | .0005 |
|  | Indirect effect of X on Y via perceived funniness | | |  |
| Mediator | *B* | SE | 95% BC CI |  |
| Perceived funniness | 0.5653 | 0.4236 | (-0.3197, 1.3478) |  |
|  | Indirect effect of X on Y via reactance | | |  |
| Mediator | *B* | SE | 95% BC CI |  |
| Reactance | -0.2574 | 0.1841 | (-0.6700, 0.0496) |  |
|  | Indirect effect of X on Y via perceived funniness and reactance | | |  |
| Mediator | *B* | SE | 95% BC CI |  |
| Perceived funniness and reactance | 0.1489 | 0.1532 | (-0.1084, 0.5098) |  |

*n* = 90

Intention: mean across three items, ranging from 1 to 7. Perceived funniness: mean across four items, ranging from 1 to 7. Reactance: mean across three items, ranging from 1 to 7. 95% BC CI: corrected 95% confidence interval with lower and upper border, based on 5,000 bootstrap resamples, CIs that do not contain zero indicate a significant indirect effect with *p* < .05.
