# Supplementary material for: Increasing the willingness to participate in organ donation through humorous health communication: (Quasi-) experimental evidence
Source: PLoS One. 2020 Nov 20;15(11):e0241208. doi: 10.1371/journal.pone.0241208 (PMC7678957; doi:10.1371/journal.pone.0241208)
Supplement: S12 Table — n = 90. Intention: mean across three items, ranging from 1 to 7. Perceived funniness: mean across four items, ranging from 1 to 7. Counter-arguing: single item, ranging from 1 to 7. 95% BC CI: corrected 95% confidence interval with lower and upper border, based on 5,000 bootstrap resamples, CIs that do not contain zero indicate a significant indirect effect with p < .05. (DOCX) [file pone.0241208.s013.docx]

S12 Table (corresponding to Figure 2B, Study 2)

*Mediation analysis: Effect of treatment (X) on intention T2 (Y) via perceived funniness (M1) and counter-arguing (M2), model 6 (Hayes, 2013).*

|  | Mediator variable model (outcome: perceived funniness) | | |  |
| --- | --- | --- | --- | --- |
| Predictor | *B* | SE | 95% CI | *p* |
| Constant | 2.3023 | 0.1922 | (1.9205, 2.6842) | <.001 |
| Treatment | 2.8892 | 0.2659 | (2.3607, 3.4176) | <.001 |
|  | Mediator variable model (outcome: counter-arguing) | | |  |
| Predictor | *B* | SE | 95% CI | *p* |
| Constant | 3.7830 | 0.3652 | (3.0572, 4.5089) | <.001 |
| Treatment | 1.5008 | 0.4767 | (0.5533, 2.4483) | .0023 |
| Perceived funniness | -0.2391 | 0.1249 | (-0.4873, 0.0091) | .0589 |
|  | Dependent variable model (outcome: intention T2) | | | |
|  | Model summary: R^2^ = 0.0837 | | |  |
| Predictor | *B* | SE | 95% CI | *p* |
| Constant | 5.1030 | 0.5581 | (3.9936, 9.2123) | <.001 |
| Treatment | -0.0644 | 0.5145 | (-1.0871, 0.9583) | .9006 |
| Perceived funniness | 0.2042 | 0.1304 | (-0.0549, 0.4634) | .1208 |
| Counter-arguing | -0.1797 | 0.1096 | (-0.3976, 0.0382) | .1048 |
|  | Indirect effect of X on Y via perceived funniness | | |  |
| Mediator | *B* | SE | 95% BC CI |  |
| Perceived funniness | 0.5901 | 0.4773 | (-0.3081, 1.4569) |  |
|  | Indirect effect of X on Y via counter-arguing | | |  |
| Mediator | *B* | SE | 95% BC CI |  |
| Counter-arguing | -0.2697 | 0.1716 | (-0.3081, 1.4569) |  |
|  | Indirect effect of X on Y via perceived funniness and counter-arguing | | |  |
| Mediator | *B* | SE | 95% BC CI |  |
| Perceived funniness and counter-arguing | 0.1241 | 0.0947 | (-0.0454, 0.3275) |  |

*n* = 90

Intention: mean across three items, ranging from 1 to 7. Perceived funniness: mean across four items, ranging from 1 to 7. Counter-arguing: single item, ranging from 1 to 7. 95% BC CI: corrected 95% confidence interval with lower and upper border, based on 5,000 bootstrap resamples, CIs that do not contain zero indicate a significant indirect effect with *p* < .05.
