# Supplementary material for: Increasing the willingness to participate in organ donation through humorous health communication: (Quasi-) experimental evidence
Source: PLoS One. 2020 Nov 20;15(11):e0241208. doi: 10.1371/journal.pone.0241208 (PMC7678957; doi:10.1371/journal.pone.0241208)
Supplement: S13 Table — n = 144. Attitude: mean across seven items, ranging from 1 to 7. Perceived funniness: mean across four items, ranging from 1 to 7. Reactance: mean across three items, ranging from 1 to 7. 95% BC CI: corrected 95% confidence interval with lower and upper border, based on 5,000 bootstrap resamples, CIs that do not contain zero indicate a significant indirect effect with p < .05. (DOCX) [file pone.0241208.s014.docx]

S13 Table (corresponding to Figure 2B, Study 2)

*Mediation analysis: Effect of treatment (X) on attitude T2 (Y) via perceived funniness (M1) and reactance (M2), controlled for the attitude T1 (covariate), model 6 (Hayes, 2013).*

|  | Mediator variable model (outcome: perceived funniness) | | |  |
| --- | --- | --- | --- | --- |
| Predictor | *B* | SE | 95% CI | *p* |
| Constant | 1.4195 | 0.7082 | (0.0194, 2.8196) | .0469 |
| Treatment | 2.9261 | 0.1975 | (2.5357, 3.3165) | <.001 |
| Attitude T1 | 0.1479 | 0.1177 | (-0.0849, 0.3806) | <.001 |
|  | Mediator variable model (outcome: reactance) | | |  |
| Predictor | *B* | SE | 95% CI | *p* |
| Constant | 6.1930 | 0.6173 | (4.0726, 7.4134) | <.001 |
| Treatment | 0.5084 | 0.2714 | (-0.0282, 1.0449) | .0631 |
| Perceived funniness | -0.0713 | 0.0724 | (-0.2144, 0.0718) | .3265 |
| Attitude T1 | -0.6895 | 0.1017 | (-0.8906, -0.4883) | <.001 |
|  | Dependent variable model (outcome: attitude T2) | | | |
|  | Model summary: R^2^ = 0.757 | | |  |
| Predictor | *B* | SE | 95% CI | *p* |
| Constant | 2.1449 | 0.3341 | (1.4843, 2.8055) | <.001 |
| Treatment | -0.1017 | 0.1134 | (-0.3260, 0.1226) | .3713 |
| Perceived funniness | 0.0410 | 0.0300 | (-0.0183, 0.1002) | .1741 |
| Reactance | -0.0816 | 0.0349 | (-0.1505, -0.0126) | .0209 |
| Attitude T1 | 0.7076 | 0.0484 | (0.6119, 0.8033) | <.001 |
|  | Indirect effect of X on Y via perceived funniness | | |  |
| Mediator | *B* | SE | 95% BC CI |  |
| Perceived funniness | 0.1199 | 0.1170 | (-0.1102, 0.3383) |  |
|  | Indirect effect of X on Y via reactance | | |  |
| Mediator | *B* | SE | 95% BC CI |  |
| Reactance | -0.0415 | 0.0360 | (-0.1285, 0.0073) |  |
|  | Indirect effect of X on Y via perceived funniness and reactance | | |  |
| Mediator | *B* | SE | 95% BC CI |  |
| Perceived funniness and reactance | 0.0170 | 0.0228 | (-0.0191, 0.0727) |  |

*n* = 144

Attitude: mean across seven items, ranging from 1 to 7. Perceived funniness: mean across four items, ranging from 1 to 7. Reactance: mean across three items, ranging from 1 to 7. 95% BC CI: corrected 95% confidence interval with lower and upper border, based on 5,000 bootstrap resamples, CIs that do not contain zero indicate a significant indirect effect with *p* < .05.
