# Supplementary material for: Increasing the willingness to participate in organ donation through humorous health communication: (Quasi-) experimental evidence
Source: PLoS One. 2020 Nov 20;15(11):e0241208. doi: 10.1371/journal.pone.0241208 (PMC7678957; doi:10.1371/journal.pone.0241208)
Supplement: S14 Table — n = 144. Attitude: mean across seven items, ranging from 1 to 7. Perceived funniness: mean across four items, ranging from 1 to 7. Counter-arguing: single item, ranging from 1 to 7. 95% BC CI: corrected 95% confidence interval with lower and upper border, based on 5,000 bootstrap resamples, CIs that do not contain zero indicate a significant indirect effect with p < .05. (DOCX) [file pone.0241208.s015.docx]

S14 Table (corresponding to Figure 2B, Study 2)

*Mediation analysis: Effect of treatment (X) on attitude T2 (Y) via perceived funniness (M1) and counter-arguing (M2), controlled for the attitude T1 (covariate), model 6 (Hayes, 2013).*

|  | Mediator variable model (outcome: perceived funniness) | | |  |
| --- | --- | --- | --- | --- |
| Predictor | *B* | SE | 95% CI | *p* |
| Constant | 1.4195 | 0.7082 | (0.0194, 2.8196) | .0469 |
| Treatment | 2.9261 | 0.1975 | (2.5357, 3.3165) | <.001 |
| Attitude T1 | 0.1479 | 0.1177 | (-0.0849, 0.3806) | .2112 |
|  | Mediator variable model (outcome: counter-arguing) | | |  |
| Predictor | *B* | SE | 95% CI | *p* |
| Constant | 7.1512 | 0.9376 | (5.2976, 9.0048) | <.001 |
| Treatment | 1.1206 | 0.4122 | (0.3056, 1.9357) | .0074 |
| Perceived funniness | -0.1958 | 0.1099 | (-0.4132, 0.0215) | .0770 |
| Attitude T1 | -0.5783 | 0.1545 | (-0.8838, -0.2727) | .0003 |
|  | Dependent variable model (outcome: attitude T2) | | | |
|  | Model summary: R^2^ = 0.7083 | | |  |
| Predictor | *B* | SE | 95% CI | *p* |
| Constant | 1.8629 | 0.3071 | (1.2557, 2.4701) | <.001 |
| Treatment | -0.1082 | 0.1165 | (-0.3385, 0.1220) | .3542 |
| Perceived funniness | 0.0407 | 0.0306 | (-0.0199, 0.1012) | .1862 |
| Counter-arguing | -0.0312 | 0.0233 | (-0.0772, 0.0148) | .1824 |
| Attitude T1 | 0.7458 | 0.0446 | (0.6575, 0.8340) | <.001 |
|  | Indirect effect of X on Y via perceived funniness | | |  |
| Mediator | *B* | SE | 95% BC CI |  |
| Perceived funniness | 0.1190 | 0.1143 | (-0.1051, 0.3401) |  |
|  | Indirect effect of X on Y via counter-arguing | | |  |
| Mediator | *B* | SE | 95% BC CI |  |
| Counter-arguing | -0.0349 | 0.0304 | (-0.0992, 0.0224) |  |
|  | Indirect effect of X on Y via perceived funniness and counter-arguing | | |  |
| Mediator | *B* | SE | 95% BC CI |  |
| Perceived funniness and counter-arguing | 0.0179 | 0.0186 | (-0.0134, 0.0609) |  |

*n* = 144

Attitude: mean across seven items, ranging from 1 to 7. Perceived funniness: mean across four items, ranging from 1 to 7. Counter-arguing: single item, ranging from 1 to 7. 95% BC CI: corrected 95% confidence interval with lower and upper border, based on 5,000 bootstrap resamples, CIs that do not contain zero indicate a significant indirect effect with *p* < .05.
