# Supplementary material for: Increasing the willingness to participate in organ donation through humorous health communication: (Quasi-) experimental evidence
Source: PLoS One. 2020 Nov 20;15(11):e0241208. doi: 10.1371/journal.pone.0241208 (PMC7678957; doi:10.1371/journal.pone.0241208)
Supplement: S15 Table — n = 86. Intention: mean across three items, ranging from 1 to 7. Perceived funniness: mean across four items, ranging from 1 to 7. Reactance: mean across three items, ranging from 1 to 7. 95% BC CI: corrected 95% confidence interval with lower and upper border, based on 5,000 bootstrap resamples, CIs that do not contain zero indicate a significant indirect effect with p < .05. (DOCX) [file pone.0241208.s016.docx]

S15 Table (corresponding to Figure 2B, Study 2)

*Mediation analysis: Effect of treatment (X) on intention T2 (Y) via perceived funniness (M1) and reactance (M2), controlled for the intention T1 (covariate), model 6 (Hayes, 2013).*

|  | Mediator variable model (outcome: perceived funniness) | | |  |
| --- | --- | --- | --- | --- |
| Predictor | *B* | SE | 95% CI | *p* |
| Constant | 1.7278 | 0.4449 | (0.8428, 2.6127) | .0002 |
| Treatment | 2.81110 | 0.2771 | (2.2598, 3.3623) | <.001 |
| Intention T1 | 0.1350 | 0.0897 | (-0.0434, 0.3133) | .1361 |
|  | Mediator variable model (outcome: reactance) | | |  |
| Predictor | *B* | SE | 95% CI | *p* |
| Constant | 3.2262 | 0.4496 | (2.3318, 4.1207) | <.001 |
| Treatment | 0.5554 | 0.3856 | (-0.2116, 1.3224) | .1535 |
| Perceived funniness | -0.0814 | 0.1020 | (-0.2844, 0.1216) | .4275 |
| Intention T1 | -0.2023 | 0.0845 | (-0.3704, -0.0343) | .0189 |
|  | Dependent variable model (outcome: intention T2) | | | |
|  | Model summary: R^2^ = 0.6895 | | |  |
| Predictor | *B* | SE | 95% CI | *p* |
| Constant | 1.8218 | 0.4283 | (0.9697, 2.6740) | .0001 |
| Treatment | -0.0133 | 0.2915 | (-0.5932, 0.5666) | .9638 |
| Perceived funniness | 0.0836 | 0.0765 | (-0.0686, 0.2358) | .2775 |
| Reactance | -0.2039 | 0.0824 | (-0.3679, -0.0399) | .0155 |
| Intention T1 | 0.7485 | 0.0652 | (0.6187, 0.8783) | <.001 |
|  | Indirect effect of X on Y via perceived funniness | | |  |
| Mediator | *B* | SE | 95% BC CI |  |
| Perceived funniness | 0.2350 | 0.2081 | (-0.1486, 0.6851) |  |
|  | Indirect effect of X on Y via reactance | | |  |
| Mediator | *B* | SE | 95% BC CI |  |
| Reactance | -0.1132 | 0.1004 | (-0.3611, 0.0208) |  |
|  | Indirect effect of X on Y via perceived funniness and reactance | | |  |
| Mediator | *B* | SE | 95% BC CI |  |
| Perceived funniness and reactance | 0.0466 | 0.0726 | (-0.0555, 0.2240) |  |

*n* = 86

Intention: mean across three items, ranging from 1 to 7. Perceived funniness: mean across four items, ranging from 1 to 7. Reactance: mean across three items, ranging from 1 to 7. 95% BC CI: corrected 95% confidence interval with lower and upper border, based on 5,000 bootstrap resamples, CIs that do not contain zero indicate a significant indirect effect with *p* < .05.
