# Supplementary material for: Increasing the willingness to participate in organ donation through humorous health communication: (Quasi-) experimental evidence
Source: PLoS One. 2020 Nov 20;15(11):e0241208. doi: 10.1371/journal.pone.0241208 (PMC7678957; doi:10.1371/journal.pone.0241208)
Supplement: S16 Table — n = 86. Intention: mean across three items, ranging from 1 to 7. Perceived funniness: mean across four items, ranging from 1 to 7. Counter-arguing: single item, ranging from 1 to 7. 95% BC CI: corrected 95% confidence interval with lower and upper border, based on 5,000 bootstrap resamples, CIs that do not contain zero indicate a significant indirect effect with p < .05. (DOCX) [file pone.0241208.s017.docx]

S16 Table (corresponding to Figure 2B, Study 2)

*Mediation analysis: Effect of treatment (X) on intention T2 (Y) via perceived funniness (M1) and counter-arguing (M2), controlled for the intention T1 (covariate), model 6 (Hayes, 2013).*

|  | Mediator variable model (outcome: perceived funniness) | | |  |
| --- | --- | --- | --- | --- |
| Predictor | *B* | SE | 95% CI | *p* |
| Constant | 1.7278 | 0.4449 | (0.8428, 2.6127) | .0002 |
| Treatment | 2.8110 | 0.2771 | (2.2598, 3.3623) | <.001 |
| Intention T1 | 0.1350 | 0.0897 | (-0.0434, 0.3133) | .1361 |
|  | Mediator variable model (outcome: counter-arguing) | | |  |
| Predictor | *B* | SE | 95% CI | *p* |
| Constant | 4.2648 | 0.5645 | (3.1418, 5.3878) | <.001 |
| Treatment | 1.4956 | 0.4841 | (0.5326, 2.4586) | .0027 |
| Perceived funniness | -0.2258 | 0.1281 | (-0.4807, 0.0291) | .0817 |
| Intention T1 | -0.1050 | 0.1061 | (-0.3161, 0.1060) | .3250 |
|  | Dependent variable model (outcome: intention T2) | | | |
|  | Model summary: R^2^ = 0.6692 | | |  |
| Predictor | *B* | SE | 95% CI | *p* |
| Constant | 1.4192 | 0.4512 | (0.5215, 2.3170) | .0023 |
| Treatment | -0.0370 | 0.3139 | (-0.6616, 0.5875) | .9063 |
| Perceived funniness | 0.0867 | 0.0801 | (-0.0727, 0.2461) | .2824 |
| Counter-arguing | -0.0598 | 0.0678 | (-0.1947, 0.0750) | .3800 |
| Intention T1 | 0.7835 | 0.0655 | (0.6532, 0.9138) | <.001 |
|  | Indirect effect of X on Y via perceived funniness | | |  |
| Mediator | *B* | SE | 95% BC CI |  |
| Perceived funniness | 0.2437 | 0.2177 | (-0.1197, 0.7420) |  |
|  | Indirect effect of X on Y via counter-arguing | | |  |
| Mediator | *B* | SE | 95% BC CI |  |
| Counter-arguing | -0.0895 | 0.1108 | (-0.3124, 0.1447) |  |
|  | Indirect effect of X on Y via perceived funniness and counter-arguing | | |  |
| Mediator | *B* | SE | 95% BC CI | *B* |
| Perceived funniness and counter-arguing | 0.0380 | 0.0550 | (-0.0663, 0.1634) |  |

*n* = 86

Intention: mean across three items, ranging from 1 to 7. Perceived funniness: mean across four items, ranging from 1 to 7. Counter-arguing: single item, ranging from 1 to 7. 95% BC CI: corrected 95% confidence interval with lower and upper border, based on 5,000 bootstrap resamples, CIs that do not contain zero indicate a significant indirect effect with *p* < .05.
