# Supplementary material for: Increasing the willingness to participate in organ donation through humorous health communication: (Quasi-) experimental evidence
Source: PLoS One. 2020 Nov 20;15(11):e0241208. doi: 10.1371/journal.pone.0241208 (PMC7678957; doi:10.1371/journal.pone.0241208)
Supplement: S17 Table — n = 144. Treatment: 0 = neutral control treatment, 1 = humorous treatment. Attitude: mean across seven items, ranging from 1 to 7. Involvement: mean across seven items, ranging from 1 to 7. 95% CI: 95% confidence interval with lower and upper border, CIs that do not contain zero indicate a significant indirect effect with p < .05. (DOCX) [file pone.0241208.s018.docx]

S17 Table

*Moderation analysis: Effect of treatment (X) on attitude T2 (Y) moderated by involvement (W), model 1 (Hayes, 2013).*

|  | Outcome variable: attitude | | | |
| --- | --- | --- | --- | --- |
|  | Model summary: R^2^ = 0.2547 | | |  |
| Predictor | *B* | SE | 95% CI | *p* |
| Constant | 5.0005 | 0.3231 | (4.3618, 5.6391) | <.001 |
| Treatment | -0.4258 | 0.4589 | (-1.3331, 0.4815) | .3551 |
| Involvement | 0.2830 | 0.0708 | (0.1430, 0.4229) | <.001 |
| Interaction: Treatment x Involvement | 0.1071 | 0.0994 | (-0.0894, 0.3037) | .2830 |

*n* = 144

Treatment: 0 = neutral control treatment, 1 = humorous treatment. Attitude: mean across seven items, ranging from 1 to 7. Involvement: mean across seven items, ranging from 1 to 7. 95% CI: 95% confidence interval with lower and upper border, CIs that do not contain zero indicate a significant indirect effect with *p* < .05.
