# Supplementary material for: Increasing the willingness to participate in organ donation through humorous health communication: (Quasi-) experimental evidence
Source: PLoS One. 2020 Nov 20;15(11):e0241208. doi: 10.1371/journal.pone.0241208 (PMC7678957; doi:10.1371/journal.pone.0241208)
Supplement: S18 Table — n = 144. Perceived funniness: mean across four items, ranging from 1 to 7. Attitude: mean across seven items, ranging from 1 to 7. Involvement: mean across seven items, ranging from 1 to 7. 95% CI: 95% confidence interval with lower and upper border, CIs that do not contain zero indicate a significant indirect effect with p < .05. (DOCX) [file pone.0241208.s019.docx]

S18 Table

*Moderation analysis: effect of perceived humour (X) on attitude T2 (Y) moderated by involvement (W), model 1 (Hayes, 2013).*

|  | Outcome variable: attitude | | | |
| --- | --- | --- | --- | --- |
|  | Model summary: R^2^ = 0.2545 | | |  |
| Predictor | *B* | SE | 95% CI | *p* |
| Constant | 4.7705 | 0.4533 | (3.8742, 5.6667) | <.001 |
| Perceived funniness | 0.0105 | 0.1098 | (-0.2066, 0.2277) | .9238 |
| Involvement | 0.3119 | 0.1000 | (0.1142, 0.5097) | .0022 |
| Interaction: Perceived funniness x Involvement | 0.0053 | 0.236 | (-0.0413, 0.0519) | .8228 |

*n* = 144

Perceived funniness: mean across four items, ranging from 1 to 7. Attitude: mean across seven items, ranging from 1 to 7. Involvement: mean across seven items, ranging from 1 to 7. 95% CI: 95% confidence interval with lower and upper border, CIs that do not contain zero indicate a significant indirect effect with *p* < .05.
