# Supplementary material for: Increasing the willingness to participate in organ donation through humorous health communication: (Quasi-) experimental evidence
Source: PLoS One. 2020 Nov 20;15(11):e0241208. doi: 10.1371/journal.pone.0241208 (PMC7678957; doi:10.1371/journal.pone.0241208)
Supplement: S19 Table — n = 90. Treatment: 0 = neutral control treatment, 1 = humorous treatment. Intention: mean across three items, ranging from 1 to 7. Involvement: mean across seven items, ranging from 1 to 7. 95% CI: 95% confidence interval with lower and upper border, CIs that do not contain zero indicate a significant indirect effect with p < .05. (DOCX) [file pone.0241208.s020.docx]

S19 Table

*Moderation analysis: Effect of treatment (X) on intention T2 (Y) moderated by involvement (W), model 1 (Hayes, 2013).*

|  | Outcome variable: intention | | | |
| --- | --- | --- | --- | --- |
|  | Model summary: R^2^ = 0.3775 | | |  |
| Predictor | *B* | SE | 95% CI | *p* |
| Constant | 1.0852 | 0.7702 | (-0.4458, 2.6163) | .1624 |
| Treatment | 1.0249 | 1.0468 | (-1.0561, 3.1058) | .3303 |
| Involvement | 0.9283 | 0.1774 | (0.5757, 1.2809) | <.001 |
| Interaction: Treatment x Involvement | -0.1662 | 0.2389 | (-0.6412, 0.3087) | .4884 |

*n* = 90

Treatment: 0 = neutral control treatment, 1 = humorous treatment. Intention: mean across three items, ranging from 1 to 7. Involvement: mean across seven items, ranging from 1 to 7. 95% CI: 95% confidence interval with lower and upper border, CIs that do not contain zero indicate a significant indirect effect with *p* < .05.
