# Supplementary material for: Increasing the willingness to participate in organ donation through humorous health communication: (Quasi-) experimental evidence
Source: PLoS One. 2020 Nov 20;15(11):e0241208. doi: 10.1371/journal.pone.0241208 (PMC7678957; doi:10.1371/journal.pone.0241208)
Supplement: S20 Table — n = 90. Perceived funniness: mean across four items, ranging from 1 to 7. Intention: mean across three items, ranging from 1 to 7. Involvement: mean across seven items, ranging from 1 to 7. 95% CI: 95% confidence interval with lower and upper border, CIs that do not contain zero indicate a significant indirect effect with p < .05. (DOCX) [file pone.0241208.s021.docx]

S20 Table

*Moderation analysis: effect of perceived humour (X) on intention T2 (Y) moderated by involvement (W), model 1 (Hayes, 2013).*

|  | Outcome variable: intention | | | |
| --- | --- | --- | --- | --- |
|  | Model summary: R^2^ = 0.4001 | | |  |
| Predictor | *B* | SE | 95% CI | *p* |
| Constant | 0.4399 | 1.0008 | (-1.5496, 2.4294) | .6613 |
| Perceived funniness | 0.3301 | 0.2282 | (-0.1235, 0.7838) | .1516 |
| Involvement | 0.9943 | 0.2333 | (0.5305, 1.4582) | <.001 |
| Interaction: Perceived funniness x Involvement | -0.0446 | 0.0524 | (-0.1488, 0.0595) | .3966 |

*n* = 90

Perceived funniness: mean across four items, ranging from 1 to 7. Intention: mean across three items, ranging from 1 to 7. Involvement: mean across seven items, ranging from 1 to 7. 95% CI: 95% confidence interval with lower and upper border, CIs that do not contain zero indicate a significant indirect effect with *p* < .05.
